# The incremental cost of improving immunization coverage in India through the Intensified Mission Indradhanush programme

**DOI:** 10.1093/heapol/czab053

**Published:** 2021-05-05

**Authors:** Susmita Chatterjee, Palash Das, Anita Pinheiro, Pradeep Haldar, Arindam Ray, Logan Brenzel, Stephen Resch

**Affiliations:** Research department, The George Institute for Global Health, Research Department, 308-309, Elegance Tower, Plot No. 8, Jasola District Centre, New Delhi 110025, India; Department of Medicine, University of New South Wales, 18 High Street, Kensington, New South Wales 2052, Australia; Prasanna School of Public Health, Manipal Academy of Higher Education, Madhav Nagar, Manipal 576104, Karnataka, India; Research department, The George Institute for Global Health, Research Department, 308-309, Elegance Tower, Plot No. 8, Jasola District Centre, New Delhi 110025, India; Research department, The George Institute for Global Health, Research Department, 308-309, Elegance Tower, Plot No. 8, Jasola District Centre, New Delhi 110025, India; Immunization Division, Ministry of Health and Family Welfare, Government of India, Nirman Bhawan, Maulana Azad Road, New Delhi 110011, India; The Bill & Melinda Gates Foundation, Capital Court, 5th Floor, Olof Palme Marg, Munirka, New Delhi 110067, India; Bill & Melinda Gates Foundation, 500 5th Ave N, Seattle, WA 98109, USA; Center for Health Decision Science, Harvard T. H. Chan School of Public Health, 718 Huntington Avenue, Boston MA 02115, USA

**Keywords:** Cost, economic cost, financial cost, incremental cost, immunization, mission Indradhanush, India

## Abstract

Intensified Mission Indradhanush (IMI) was a strategic endeavour launched by the Government of India aiming to achieve 90% full immunization coverage in the country by 2018. The basic strategy of this special drive involved identifying missed children and vaccinating them in temporary outreach sites for 1 week over consecutive 4-month period starting from October 2017. This study estimated the incremental economic and financial cost of conducting IMI in India from a government provider perspective. Five states—Assam, Bihar, Maharashtra, Rajasthan and Uttar Pradesh were purposefully selected because of high concentration of IMI activities. The stratified random sample of 40 districts, 90 sub-districts and 289 sub-centres were included in this study. Cost data were retrospectively collected at all levels from administrative records, financial records and staff interviews involved in IMI. The weighted incremental economic cost per dose (including vaccine costs) was lowest in Uttar Pradesh (US$3.45) and highest in Maharashtra (U$12.23). Incremental economic cost per IMI dose was found to be higher than a recent routine immunization costing study by Chatterjee and colleagues in 2018, suggesting that it requires additional resources to immunize children through an intensified push in hard-to-reach areas. Incremental financial cost of the IMI programme estimated in this study will be helpful for the government for any future planning of such special initiative. The reasons for variation of unit costs of IMI across the study districts are not known, but lower baseline coverage, high population density, migration, geography and terrain and vaccinating small numbers of children per session could account for the range of findings. Further analysis is required to understand the determinants of cost variations of the IMI programme, which may aid in better planning and more efficient use of resources for future intensified efforts.

**Key messages**

- Recent literature reviews indicated that several studies in high-, low- and middle-income countries have reported the improvement in immunization coverage through different interventions; however, the majority of those did not report the incremental costs of these interventions, which is a missed opportunity. In India, there is only one study that reported cost analysis of an intervention aimed to improve immunization coverage in one district.
- This study estimated incremental financial and economic cost of conducting Intensified Mission Indradhanush (IMI) programme, a Government of India initiative to improve immunization coverage of the country, by covering 40 districts, 90 sub-districts and 289 sub-centres across five states in India.
- Incremental economic cost per dose delivered through IMI programme was higher than economic cost per dose estimated in a recent routine immunization costing study in India, suggesting that it requires additional resources to immunize children through an intensified push in hard-to-reach areas.
- Time cost was the major cost contributor, and a significant proportion of the time was spent for conducting IMI sessions itself. A better planning of the programme could reduce the cost. Incremental financial cost of IMI programme estimated in this study will be helpful for the government for any future planning of such special initiative.

## Introduction

Intensified Mission Indradhanush (IMI) was a Government of India initiative to improve immunization coverage of the country. Despite being operational for about 35 years, India’s universal immunization programme was only able to fully immunize up to 62% of the eligible children in 2015–16 (International Institute for Population Sciences (IIPS) and ICF, 2017). To increase the coverage to 90% by 2018, IMI, the programme of periodic intensification of routine immunization, was implemented by the government in 173 lower-coverage districts and 17 urban areas across 24 states during October 2017 to January 2018 (Gurnani *et al.*, 2018). The programme targeted all children aged up to 5 years and the pregnant women with a focus on full immunization of children under 2 years. The basic strategy was based on a head count survey to identify children with missing doses, resulting in a due list of missing children, and preparation of a microplan for conducting sessions during 1 week of each month for four consecutive months in new vaccination sites. If required, mobile teams were formed to reach large mobile and isolated population (e.g. population in brick kilns, construction areas and plantation areas). The objective of forming the mobile teams was to conduct the outreach sessions in these hard-to-reach areas at convenient time and places. All categories of staff were trained before the implementation of the programme, and the programme was regularly monitored at different levels (Gurnani *et al.*, 2018).

Ninety seven thousand six hundred and twenty-eight vaccination sessions were conducted in IMI during October 2017 and January 2018, where 5.95 million children and 1.18 million pregnant women were vaccinated. Over 15 million antigens were delivered during this period. Gurnani *et al.*, (2018) described the programme in detail and its impact on immunization coverage of the country. They found that after IMI, proportion of children with full immunization coverage increased by 18.5% as compared with pre-IMI estimates. However, they concluded that the whole increase in coverage was not solely because of IMI—other similar initiatives also influenced the improvement. Another study conducted a quasi-experimental evaluation of IMI by comparing IMI and non-IMI districts using routine administrative data on vaccine doses delivered (Clarke-Deelder *et al.*, 2021). This controlled interrupted time-series analysis estimated the impact of IMI during the 4-month implementation period and in subsequent months. The study found that during implementation, IMI increased the delivery of vaccine doses with a median effect of 10.6%; however, there was no evidence of a sustained effect of IMI during 8 months after the implementation ended. It is evident from both studies that IMI had some impact on immunization coverage, and it is obvious that a special drive like IMI required additional resources. The objective of this study was to estimate the incremental financial as well as economic cost of conducting IMI programme.

## Methods

A government provider perspective was used to calculate the incremental economic and financial cost of conducting IMI programme (Brenzel *et al.*, 2015). The IMI began in October 2017 and continued until January 2018. Cost data were retrospectively collected by a 5-member team between July 2018 and January 2019 using standardized and pre-tested questionnaires. All costs were presented in 2019 US$. An average exchange rate of 2019, US$1 = INR 70.394, was used throughout the paper.

### Sampling methodology

Five states with high concentration of IMI activities (Assam, Bihar, Maharashtra, Rajasthan and Uttar Pradesh) were purposefully selected for this study. Assam is a north-eastern state in India with a population of approximately 31 million as per 2011 census. Bihar, the third populous and the most densely populated state, situated in eastern India had a population of approximately 104 million in 2011. Maharashtra, a western India state, had a population of 112 million in 2011 and is the second populous state in India. Rajasthan and Uttar Pradesh are states in northern India. As per 2011 census, Rajasthan had about 68 million population while Uttar Pradesh had roughly 200 million inhabitants, which makes the state the most populous state in the country. Full immunization coverage as per National Health and Family Welfare Survey (2015–2016) was the lowest in Assam (47.1%), followed by Uttar Pradesh (51.1%), Rajasthan (54.8%), Maharashtra (56.2%) and Bihar (61.7%) (International Institute for Population Sciences (IIPS) and ICF, 2017). Because of huge numbers of unvaccinated children, majority of IMI activities were concentrated in these five states.

Within these five states, 34 districts where IMI programme was conducted were randomly selected for this study. During the sampling process, first, all IMI districts in five study states were grouped by division, and divisions were selected at random using probability proportional to size sampling. Up to two IMI districts per division were randomly selected. The number of sub-districts (blocks) selected per district was based on a rule of selecting 30% of sub-districts, with a floor of two sub-districts and a ceiling of four sub-districts. The number of sub-centres (SCs) per sub-district was based on a rule of selecting 10% of SCs, with a floor of two and a ceiling of four SCs. The rule of selecting sub-districts and SCs was to manage the total size of the sample to fit the data collection timeframe and budget.

In addition to 34 districts, six districts with urban areas were also selected for this study as improving immunization coverage in urban areas was a major focus of IMI programme. In Uttar Pradesh and Maharashtra, districts with urban areas were selected at random, in Bihar and Rajasthan, IMI was conducted in one district each having urban areas and those two districts were selected for this study. Assam did not have any district with urban areas for IMI.

The final sample consisted 34 randomly selected IMI districts and six other IMI districts with urban areas, 90 sub-districts (about 30% of all sub-districts), 239 SCs and 50 urban auxiliary nurse midwives (ANMs) (about 3% of all SCs). In Indian health system, SCs are the most peripheral and first point of contact between the primary health care system and the community. In the field of rural health, one SC covers approximately 5000 population in plains and is managed by 1–2 ANMs who are primarily responsible for routine immunization in their respective areas. During IMI, they were responsible for head count survey, due list and microplan preparation and conducting sessions. In urban areas, as there are no sub-districts or SCs, ANMs are posted either in the urban health post or in urban primary health centre. Three to four such urban units were randomly selected, followed by random selection of 2–3 ANMs in those units. The distribution of final sample is given in Table 1.

**Table 1.** Study sample

State	Districts	Districts with urban areas	Sub-districts	SCs	ANMs in urban areas
Assam	5	0	10	28	0
Bihar	6	1	18	40	7
Maharashtra	5	1	18	54	8
Rajasthan	5	1	12	22	8
Uttar Pradesh	13	3	32	95	27
Total	34	6	90	239	50

ANM, Auxiliary Nurse Midwives.

### Costing methodology

Both economic and financial costs were calculated to understand the incremental cost of conducting IMI. Costs were calculated based on an internationally recognized and standardized approach, adapted for this study (Brenzel *et al.*, 2015). The unit of incremental cost analysis was the district as IMI was conducted at the low-performing districts only. At the district and sub-district levels, the activities related to IMI included task force and routine review meetings, training, supervision, report compilation, microplan compilation, vaccine distribution and social engagement. At the SC/ANM level, activities comprised the head count survey, due list and microplan preparation, travel to the session sites, conducting the sessions and report preparation.

Data were collected at the district and sub-district levels regarding the quantity, duration and number of participants in each activity. Sources of data included training and meeting registers, the supervision plan, the microplan for routine immunization and IMI. At the SC level, ANMs were interviewed to understand the staffing of the IMI sessions and time spent for different activities related to IMI. At the district and sub-district levels, antigen-wise doses delivered during IMI were gathered from administrative records.

Additional expenditure on vaccine transport, communication (all activities to inform the community about IMI, e.g. banners, posters, miking and media coverage), training, meeting, mobility support, payment for alternate vaccine delivery (AVD)^1^, incentives for Accredited Social Health Activists (ASHAs)^2^, printing, waste management, supervision, microplanning and mobile team were gathered from the financial reports of each sampled district and were included in both economic and financial cost calculation. Travel expenses to session sites were gathered from interviews of the ANMs and were also added into both cost calculations.

Vaccine and syringe costs were calculated by multiplying number of doses of different antigens administered during IMI, wastage rate and unit price and were added in both financial and economic cost calculations. Antigen-wise unit price, unit price of syringes and wastage rates were taken from the cost analysis of India’s comprehensive multi-year plan for immunization (2018–2022) (Ministry of Health and Family Welfare, Government of India) and are presented in Supplementary Appendix Table A1.

In addition to the cost items mentioned above, time spent on each activity was multiplied by respective hourly wage to calculate the time cost related to that activity, which was part of economic cost of IMI. At the district and sub-district levels, monthly gross salary of each staff involved in IMI activities was collected from the pay roll division. Monthly working hours and gross monthly salary was used to calculate the hourly wage for each staff. Average monthly gross salary of different categories of staff involved in IMI programme is presented in Supplementary Appendix Table A2. Time spent in various IMI activities was collected through face-to-face interviews with staff involved in IMI activities. For calculating the time spent by voluntary workers (e.g. ASHAs), minimum wage rate of the respective states was used.

The goal of collecting cost data from a sample of health facilities was to make inferences of unit cost and total costs of the IMI programme in the sampled districts and states. To minimize bias and maximize precision in unit cost estimates, volume-weighted mean of unit costs across the sites in the sample was used, which is a recommended method for healthcare costing studies in low- and middle-income countries (Clarke-Deelder *et al.*, 2019). The volume-weighted mean unit cost was calculated as the sum of the total costs across all sites in the sample divided by the sum of delivery volumes (number of doses administered during IMI; number of children vaccinated during IMI) across all sites in the sample.

The uncertainty in estimating cost comes from the uncertainty due to selection of sample facilities at different levels. One approach to obtain a correct estimate is to use inverse probability weighting (Rivera-Rodriguez *et al.*, 2018). Weights are the inverse of the probability of a sample to be selected from the total (Resch *et al.*, 2020). To calculate the uncertainty, the variance of the weighted estimate of total costs was calculated using the methods discussed by Rivera-Rodriguez *et al.*, (2018). After deriving the variance, the confidence interval (CI) was calculated using the standard formula:

$$\begin{equation*}CI = mean \pm z{{sd} \over {\sqrt n }}\end{equation*}$$

where *z* is the *z*-score corresponding to the confidence level (95%), *sd* is the standard error, the square root of variance, and *n* is the sample size.

## Results

### IMI outputs

Considering the study districts of five study states, the incremental average number of doses delivered during all sessions in IMI ranged from 14 353 in Assam to 183 471 in Uttar Pradesh. Similar variation was observed for children vaccinated during IMI. Average children vaccinated during IMI in the study districts was 3784 in Assam; 6219 in Rajasthan; 7690 in Maharashtra; 17 974 in Bihar and 66 619 in Uttar Pradesh.

Over the 4-month period of IMI, sampled ANMs in Maharashtra conducted four sessions (1 session per ANM per month); ANMs in Assam conducted six IMI sessions; in Rajasthan eight sessions; in Bihar 10 sessions; and in Uttar Pradesh, 24 sessions on average. These IMI sessions were incremental to ANMs’ ongoing routine immunization sessions.

### Economic cost of IMI

#### Time spent and time cost for IMI at the SC level

At the SC level, most of the time was spent for conducting the IMI session itself. Average duration of each session was 7 hours excluding travel time. The average round trip travel time from the respective SC to the IMI session site was 71 minutes for Assam ANMs; 57 minutes for Bihar ANMs; 23 minutes for Maharashtra ANMs; 27 minutes for Rajasthan ANMs and 26 minutes for Uttar Pradesh ANMs. Motorcycle was the most common mode of transport used for reaching the session sites (53% session sites were reached by motorcycle) followed by walking (18% session sites), office vehicle (10%) and auto rickshaw (9%). In districts with urban areas, ANMs conducted more sessions during IMI except in Uttar Pradesh and the number ranged from 8 to 22 sessions. Travel time to the session sites was less in urban areas of Bihar and Maharashtra but was similar in Rajasthan and Uttar Pradesh. In the urban areas, the main transport to reach the session sites was auto rickshaw/rickshaw (covered 43% sessions sites). The other modes of transport were walking (in 27% session sites), motorcycle (17%) and office vehicle (9%).

**Figure 1.**
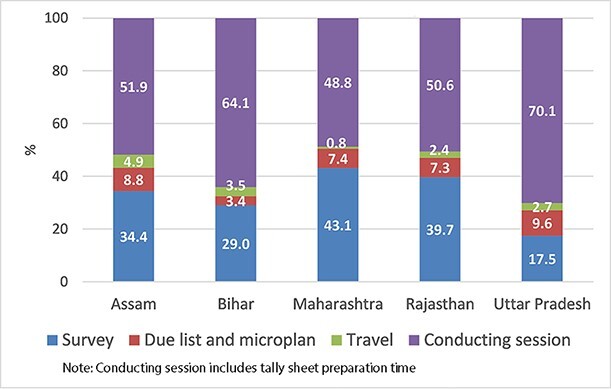
Distribution of labour hours related to Intensified Mission Indradhanush (IMI) at the sub-centre level (average %).

During the IMI sessions, ANMs were assisted by ASHAs and other staff members. The majority were present full time during the sessions, which increased total hours spent for IMI sessions. Total hours spent for all activities related to IMI at the SC level varied widely across study states, with the fewest hours spent in Assam (291 hours total) and the highest in Uttar Pradesh (752 hours total). In Rajasthan SCs, total 331 hours were spent for IMI activities followed by 390 hours in Maharashtra SCs and 433 hours in Bihar SCs. The percentage distribution of labour hours related to various IMI related activities at the SC level is presented in Figure 1. Most of labour hours were spent for conducting IMI sessions ranging from 49% in Maharashtra to 70% in Uttar Pradesh (Figure 1). After conducting session, a significant proportion of labour hours were spent for conducting the head count survey. In Maharashtra and Rajasthan, the survey accounted for 43% and 40% of total labour hours, respectively. District-wise distribution of time costs for various IMI activities at the SC level is presented in Supplementary Appendix Table A3.1

#### Time cost at the sub-district and district levels

The majority of labour time cost at the district and sub-district levels was for training and meeting related to IMI (48% of total time cost both at district and sub-district levels) and extensive supervision of IMI (46% at the sub-district level and 49% at the district level). Report compilation contributed about 2% of total time cost at both levels. Vaccine distribution contributed about 3% of total time cost at the sub-district level; however, at the district level, there was no additional time cost related to vaccine distribution because during IMI, vaccines were transported and distributed along with routine vaccines. District-wise distribution of time costs related to IMI activities at the sub-district and district levels is presented in Supplementary Appendix Tables A3.2 and A3.3, respectively.

#### Incremental economic cost of conducting IMI

The incremental economic cost per IMI dose and cost per child vaccinated during IMI varied substantially across the study states and districts. Number of doses were antigen-wise total doses delivered during IMI period and were gathered from administrative records. As one child might get more than one antigen in one visit (e.g. first dose of pentavalent, oral polio and rotavirus vaccine), number of doses were much higher than the number of children vaccinated during IMI sessions. Incremental economic cost of IMI was divided by total number of doses and total children vaccinated during IMI period to get cost per IMI dose and cost per child vaccinated, respectively. Unit costs were the lowest in Uttar Pradesh and highest in Maharashtra (Table 2). Among all 40 study districts, the lowest cost per dose was in Patna district of Bihar (US$1.73) and highest in Nanded district of Maharashtra (US$23.26) (Table 3).

**Table 2.** Weighted incremental economic cost (including vaccine cost) of conducting IMI programme (US$2019)

State	Incremental economic cost (US$ million)	95% CI (LB, UB)	Cost per IMI dose ($2019)	95% CI (LB, UB)	Cost per child vaccinated during IMI ($2019)	95% CI (LB, UB)
Assam	0.49	0.43, 0.55	6.75	5.99, 7.50	24.61	21.78, 27.44
Bihar	4.18	3.15, 5.21	4.73	2.83, 6.63	12.06	7.61, 16.51
Maharashtra	2.68	1.93, 3.43	12.23	7.23, 17.23	28.24	16.75, 39.72
Rajasthan	1.25	0.96, 1.55	5.83	4.62, 7.04	18.32	14.59, 22.05
Uttar Pradesh	40.08	37.02, 43.15	3.45	2.86, 4.03	9.22	7.54, 10.91

CI, confidence interval; LB, lower bound; UB, upper bound.

**Table 3.** Weighted incremental economic cost (including vaccine cost) of conducting IMI programme in study districts (US$2019)

District	Incremental economic cost (US$ million)	95% CI (LB, UB)	Doses delivered during IMI^a^	Cost per IMI dose	95% CI (LB, UB)	Children vaccinated during IMI	Cost per child vaccinated during IMI	95% CI (LB, UB)
Assam								
Chirang	0.04	0.038, 0.045	3350	12.36	11.28, 13.43	947	43.71	39.90, 47.53
Darrang	0.05	0.047, 0.060	12 384	4.35	3.83, 4.88	3371	15.99	14.06, 17.92
Karbi Anglong	0.11	0.094, 0.123	17 873	6.08	5.26, 6.90	5107	21.29	18.42, 24.16
Kokrajhar	0.05	0.048, 0.059	8559	6.21	5.57, 6.85	2374	22.39	20.10, 24.69
Nagaon	0.14	0.124, 0.156	29 600	4.73	4.18, 5.28	7119	19.67	17.38, 21.95
Bihar								
East Champaran	0.17	0.171, 0.175	39 974	4.32	4.27, 4.38	15 191	11.38	11.23, 11.53
Gaya	0.56	0.476, 0.644	75 270	7.44	6.33, 8.55	28 812	19.44	16.54, 22.34
Madhubani	0.44	0.404, 0.477	68 269	6.45	5.92, 6.99	34 020	12.95	11.87, 14.02
Nawada	0.08	0.070, 0.090	22 040	3.61	3.16, 4.07	7953	10.01	8.75, 11.28
Patna (urban)	0.09	0.085, 0.094	51 681	1.73	1.65, 1.82	20 254	4.42	4.20, 4.64
Sheohar	0.05	0.038, 0.061	15 616	3.17	2.46, 3.87	5973	8.28	6.43, 10.13
Sitamarhi	0.24	0.226, 0.263	38 368	6.37	5.89, 6.85	13 615	17.95	16.60, 19.30
Maharashtra								
Ahmednagar	0.17	0.127, 0.211	11 717	14.42	10.84, 18.02	4971	34.00	25.52, 42.49
Beed	0.10	0.096, 0.113	11 970	8.74	8.02, 9.46	4448	23.52	21.58, 25.46
Jalgaon	0.40	0.315, 0.479	22 658	17.51	13.90, 21.23	10 216	38.84	30.82, 46.87
Nanded	0.20	0.157, 0.243	8588	23.26	18.28, 28.24	3913	51.05	40.12, 61.98
Solapur	0.13	0.103, 0.156	21 800	5.96	4.75, 7.18	9639	13.48	10.73, 16.23
Thane (urban)	0.11	0.086, 0.135	31 751	3.48	2.70, 4.26	12 953	8.53	6.62, 10.44
Rajasthan								
Alwar	0.13	0.119, 0.141	8015	16.22	14.83, 17.61	2541	51.15	46.77, 55.54
Jaipur (urban)	0.07	0.052, 0.094	17 504	4.15	2.94, 5.36	6425	11.32	8.02, 14.61
Jodhpur	0.14	0.089, 0.183	44 567	3.06	2.00, 4.12	14 408	9.46	6.20, 12.73
Pali	0.09	0.079, 0.099	17 375	5.13	4.55, 5.72	5320	16.77	14.86, 18.69
Pratapgarh	0.02	0.010, 0.033	7557	2.90	1.36, 4.43	2446	8.95	4.21, 13.69
Udaipur	0.08	0.042, 0.110	21 387	3.54	1.95, 5.13	6172	12.27	6.77, 17.78
Uttar Pradesh								
Bahraich	1.04	0.982, 1.105	456 742	2.28	2.15, 2,42	148 917	7.01	6.59, 7.42
Ballia	0.88	0.807, 0.955	204 078	4.32	3.95, 4.68	79 021	11.15	10.21, 12.08
Balrampur	0.51	0.490, 0.528	195 015	2.61	2.51, 2.71	65 358	7.79	7.49, 8.08
Banda	0.52	0.487, 0.556	110 446	4.72	4.41, 5.03	42 370	12.30	11.49, 13.12
Basti	0.63	0.530, 0.729	152 737	4.12	3.47, 4.77	59 507	10.58	8.91, 12.26
Chitrakoot	0.22	0.175, 0.261	59 794	3.64	2.92, 4.36	23 076	9.43	7.57, 11.30
Farrukkabad	0.53	0.469, 0.588	169 658	3.11	2.76, 3.46	65 911	8.01	7.11, 8.92
Hapur	0.28	0.205, 0.361	72 187	3.92	2.84, 5.00	28 378	9.96	7.21, 12.72
Hardoi	0.98	0.908, 1.053	291 228	3.37	3.12, 3.62	100 608	9.74	9.02, 10.46
Jaunpur	1.43	1.293, 1.558	351 853	4.05	3.67, 4.43	123 907	11.50	10.44, 12.57
Lucknow (urban)	0.39	0.276, 0.497	130 855	2.95	2.11, 3.80	47 920	8.06	5.75, 10.37
Mau	0.64	0.600, 0.676	148 528	4.30	4.04, 4.55	60 357	10.57	9.94, 11.20
Meerut (urban)	0.16	0.146, 0.168	81 678	1.92	1.79, 2.06	35 672	4.41	4.10, 4.71
Sidharthnagar	0.80	0.723, 0.874	219 430	3.64	3.30, 3.98	70 628	11.31	10.24, 12.37
Unnao	0.81	0.769, 0.842	205 346	3.92	3.75, 4.10	75 336	10.69	10.21, 11.17
Varanasi (urban)	0.20	0.171, 0.224	85 957	2.30	1.99, 2.60	38 939	5.07	4.39, 5.74

CI, Confidence Interval; LB, Lower bound; UB, Upper bound.

*Note*: 1US$ = INR 70.394.

a Including tetanus toxoid doses.

### Financial cost of IMI

**Figure 2.**
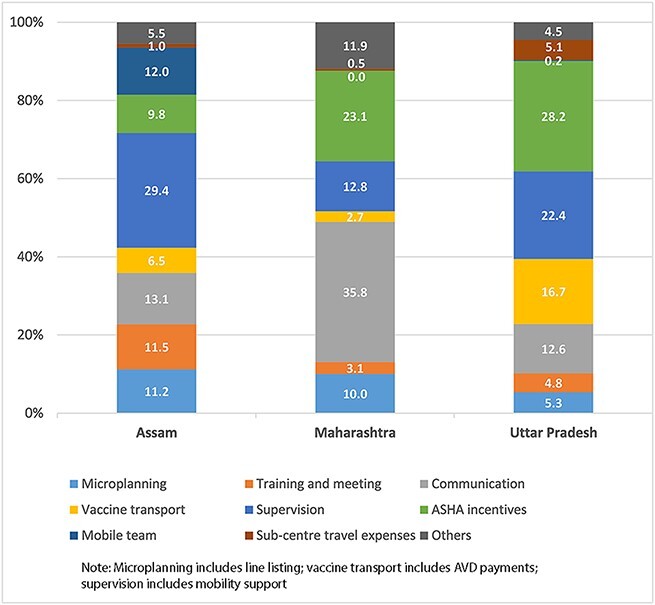
Distribution of financial cost of Intensified Mission Indradhanush (IMI) (average % - excluding vaccines and syringes) in selected states.

At the district level, on average, financial cost for IMI was about 24% of incremental economic cost of conducting IMI, which indicates the labour-intensive nature of the intervention. Vaccines and syringes accounted for the major share of financial cost (average ranging between 60% and 78%) for the sampled IMI districts in three states: Bihar, Rajasthan and Uttar Pradesh. In the sampled IMI districts of Assam and Maharashtra, vaccines and syringes represented on average 29% and 25% of financial costs, respectively. The distribution of financial cost of IMI for the sampled districts in three states is presented in Figure 2. Detailed distribution of financial cost across the districts of Assam, Maharashtra and Uttar Pradesh are presented in Supplementary Appendix Table A4. For few districts of Bihar and Rajasthan, component-wise financial expenditure data were not available; therefore, distribution of financial cost was not presented for these two states.

#### Incremental financial cost of conducting IMI

Incremental financial cost of IMI also varied across study states and study districts; however, the variation was not as wide as incremental economic cost. Weighted-average financial cost per dose was lowest in Rajasthan and Uttar Pradesh and highest in Assam (Table 4). Assam also had highest financial cost per child vaccinated during IMI.

**Table 4.** Weighted incremental financial cost (including vaccine cost) of conducting IMI programme (US$2019)

State	Incremental financial cost (US$ million)	95% CI (LB, UB)	Cost per IMI dose ($2019)	95% CI (LB, UB)	Cost per child vaccinated during IMI ($2019)	95% CI (LB, UB)
Assam	0.17	0.15, 0.18	2.25	2.03, 2.46	8.16	7.35, 8.98
Bihar	0.82	0.67, 0.97	1.06	0.78, 1.33	2.72	2.07, 3.36
Maharashtra	0.39	0.30, 0.49	1.57	0.99, 2.16	3.67	2.32, 5.03
Rajasthan	0.22	0.15, 0.28	0.78	0.50, 1.06	2.43	1.56, 3.30
Uttar Pradesh	9.54	8.66, 10.42	0.78	0.48, 1.08	2.11	1.25, 2.97

CI, confidence interval; LB, lower bound; UB, upper bound.

## Discussion

This paper estimated the incremental financial and economic cost of conducting IMI, a flagship programme of the government of India, aimed to improve immunization coverage of the country. Time cost contributed 63–83% of total incremental economic cost of conducting IMI indicating the labour-intensive nature of the intervention. A study calculated weighted economic costs of routine immunization across seven states in India including Bihar and Uttar Pradesh where the current study was also conducted (Chatterjee *et al.*, 2018). Incremental economic cost per IMI dose was substantially higher than the same estimated for routine immunization in these two states. The cost per dose reported for routine immunization was US$1.31 for Bihar and US$1.43 for Uttar Pradesh in 2019 US$. Incremental cost per IMI dose was US$4.73 in Bihar and US$3.45 in Uttar Pradesh in 2019 US$. The higher unit cost in IMI was because IMI aimed to reach the unreached and hence, a lot more effort was given for identifying the missed children through extensive household survey, several planning meetings were conducted to finalize additional temporary session sites for vaccinating those missed children. Children were vaccinated in hard to reach areas such as riverine islands, which remain inaccessible half of the year, and in areas dominated by migrant workers such as brick kiln, construction sites and plantation. Because of these extensive efforts to reach to the last mile for vaccination, time cost of the special drive was significantly higher, and hence, the incremental economic cost.

During the interviews with 289 sampled ANMs, they were asked ‘in your experience how many kids is the most you can vaccinate in an hour in routine setting’, i.e. in any routine immunization session (fixed or outreach) if kids are taken at sites and wait in queue, how many they can vaccinate per hour. The ANMs reported they could vaccinate on average 13 kids per hour in a typical routine immunization session while on average, only around two kids were vaccinated per hour during IMI. The average time spent by the ANMs and accompanied staff members per IMI session was 7 hours, which increased the time cost substantially. A better planning of the programme could have reduced the cost. Adjusting the duration of the IMI session based on ANMs’ perception of number of children that could be vaccinated per hour, the incremental cost per dose of IMI decreased by 11–32% in the study states. In geographies with a few numbers of children to be vaccinated during IMI sessions, these children could have been vaccinated during routine sessions with the exception of the hard-to-reach areas. If it was absolutely required to reach those children through IMI, then the ANMs could have had greater autonomy to decide the timing and duration of the sessions. Several sampled ANMs reported that even when they had few kids to vaccinate as per due list, they had to wait at the session site for the entire planned period of time (on average 6–7 hours), sometimes without proper infrastructure and facilities. Proper utilization of their time will not only reduce the incremental cost of the special drive like IMI, but health workers’ time can be utilized for other productive activities. During any future programme planning like IMI, the authorities should also look into the safety of the ANMs (e.g. while planning vaccination at the construction sites or in forest areas, ANMs should be accompanied by at least another staff member) and ensure that minimum infrastructure (e.g. tables, chairs and a proper place to sit) and facilities (e.g. toilet facilities) are available at the session sites. Gurnani *et al.*, (2018) also noted lack of infrastructure as one of the challenges in new session sites during IMI. These are particularly important in the coronavirus disease-2019 (COVID-19) era when alongside the safety of the vaccination team, the children and their parents, maintaining and improving the coverage will also be a challenge.

Recent literature reviews indicated that several studies in high-, low- and middle-income countries have reported the improvement in immunization coverage through different interventions. However, the majority of those did not report the incremental costs of these interventions, which is a missed opportunity (Ozawa *et al.*, 2018; Munk *et al.*, 2019). Without cost information, countries will not have enough evidence on the additional resources needed to achieve performance goals. The literature review identified four studies in India that reported either total intervention cost for improving immunization coverage or unit costs (Pandey *et al.*, 2007; Rainey *et al.*, 2009; Banerjee *et al.*, 2010; Powell-Jackson *et al.*, 2018); however, only one study reported the contribution of different cost components in total cost of the intervention. Banerjee *et al.*, (2010) assessed the efficacy of non-financial incentives on immunization rates and compared it with the effect of improving the reliability of the supply of services only. Reliability of supply of services implied regular availability of immunization services in the sampled villages through conducting mobile immunization camps on a fixed date every month at a fixed time. This was compared with another intervention where in addition to ensuring availability of immunization services through monthly immunization camps, parents were offered non-financial incentives per immunization administered and on completion of child’s full immunization. The average cost of a fully immunized child was US$28 in reliable camps with incentive and US$56 in camp without incentive. The largest cost component was salaries (29%) followed by incentives (28%) and monitoring (23%) for the intervention where incentives were given for immunizing a child. Salary contributed 46% of total cost for the intervention focused only on improving the reliability of supply of services followed by monitoring (37%). Salary was the largest cost component because staff was hired specifically for the intervention. In IMI programme, staff contributed a proportion of their time for IMI activities but still time cost contributed to 63–83% of total incremental economic cost of conducting IMI, indicating that much more effort was required to reach out the missed-out children. This is obvious because during IMI, ANMs reached out to several hard-to-reach areas such as riverine islands, brick kiln and construction sites and plantation areas and because of such extensive effort to reach out to the last mile, time cost was significantly high.

Apart from immunization, ANMs are also responsible for family planning, antenatal check-ups, delivery, distribution of zinc tablets and oral rehydration solution packets. ANMs were asked whether they missed such activities during IMI because of regular IMI sessions in 1 week for consecutive 4 months and whether their missed work was conducted by someone else. The majority ANMs admitted that even if they missed some routine activities, they had to cover those after the IMI week was over, as there was no substitute to perform their missed work. It is well recognized that Indian health system runs with severe shortage of manpower (Karan *et al.*, 2019; World Health Organization, 2021). Proper planning of IMI sessions could help deploy and utilize staff time more effectively. It would also be interesting to examine whether other services delivered by ANMs were affected because of IMI.

While comparing the routine immunization microplan before and after IMI, it was noted that there was a change in routine microplan for only one ANM out of 28 sampled ANM in Assam. In most of the sampled IMI districts in Assam, because of the seasonality of brick kiln activities and inaccessibility of riverine areas, the ANMs utilized the IMI programme to reach those unreached pockets. Because of uncertainty in reaching the population groups, it is difficult for them to include these areas under their routine microplan; hence, minimum change in their routine microplan was noticed after IMI. At the same time, it is equally important to reach out to these population whenever they are accessible. It will be the ANMs’ initiatives to ensure that children in such hard-to-reach areas are vaccinated.

The low percentage change in routine microplan after IMI in other study states raises the concern about the sustainability of the effects of the IMI initiative. Even though several ANMs had mentioned that because of strong motivation during IMI, children started coming in routine sessions after IMI, this needs to be investigated further. A recent study used a quasi-experimental approach to estimate the effects of IMI during the implementation period and subsequent 8 months to examine whether the effects of IMI sustained after implementation ended (Clarke-Deelder *et al.*, 2021). The study found that during IMI implementation there was improvement of immunization coverage; however, the improvement did not sustain after implementation. This finding is in line with the current study finding of low percentage change in routine immunization microplan. This implies that efforts are required to ensure that the additional children vaccinated during a special drive like IMI are coming into the routine immunization sessions.

### Limitations

The following limitations of the study need to be noted. First, our data collection began 6 months after IMI was conducted. This could result in recall bias on the time spent for different activities related to IMI. To minimize this, whenever possible, additional evidence was collected to verify time spent. For example, in a few training and meeting registers, information was available at the start and end time of the event, along with number of participants and proceedings. Given that ANMs and their supervisors adhered to a schedule for duration of IMI sessions, time spent for conducting sessions and supervision was relatively robust, and recall bias would be minimized. Second, given the size of the cost study, there were gaps in information to be collected. For instance, Pratapgarh and Udaipur districts of Rajasthan did not include SC cost data as the ANMs were on indefinite strike during data collection. Actual financial expenditure for the IMI was not available for Patna district of Bihar but was imputed by an average of similar districts’ financial expenditure. Data collection was also not possible from two sampled sub-districts in East Champaran district of Bihar because of strike of ASHAs. In urban areas, a few activities during IMI (e.g. meeting) might have happened at the urban health units. As the records of such activities were not properly maintained in some of those units, the team was unable to collect detailed data. Finally, our analysis excludes the value of time spent of non-health sector staff, such as participants from the education department.

## Conclusion

The IMI was a Government of India initiative to improve immunization coverage in the country and it was solely funded by the government. In the sampled states, the incremental economic cost of the IMI ranged from US$0.49 million to US$40.08 million, with most of the cost at the SC level. The incremental economic cost per IMI dose was found to be higher than a recent routine immunization costing study, suggesting that it requires additional resources to immunize children through an intensified push in hard-to-reach areas. To our knowledge, this is one of the first and largest studies that document the incremental costs of scaling up immunization coverage through such an intensified effort. This analysis also showed a wide variation of the costs across states and within states. The reasons of variation of unit costs of IMI across the study districts need to be evaluated further, but lower baseline coverage, high population density, geography and terrain and vaccinating small numbers of children per session could account for the range of findings. Further analysis is required to understand the determinants of cost variations of the IMI programme, which may aid in better planning and more efficient use of resources for future intensified efforts.

## Supplementary Material

czab053_Supp

## Data Availability

Data for this study will be publicly available on the Harvard EPIC DataVerse Repository.

## Endnotes

^1^
Alternate vaccine delivery (AVD) is a vaccine delivery system introduced by the government to deliver vaccines from the nearest cold chain point to immunization session sites in each session day. During IMI as well as routine immunization sessions, the person responsible for AVD received US$1.1 per session and US$2.2 per session in hard-to-reach areas for delivering vaccines.

^2^
An ASHA is a trained female community health worker acts as an interface between the public health system and community. Performance-based incentives are given to ASHAs for different activities including immunization. During IMI, ASHAs received incentives for preparing due list of children, mobilizing the children at session sites.
