# Supplementary material for: The incremental cost of improving immunization coverage in India through the Intensified Mission Indradhanush programme
Source: Health Policy Plan. 2021 May 5;36(8):1316–24. doi: 10.1093/heapol/czab053 (PMC8428614; doi:10.1093/heapol/czab053)
Supplement: czab053_Supp [file czab053_supp.zip › Appendix_Table_Revised.docx]

**Table A1: Unit price and wastage rates of vaccines and syringes**

| Name of vaccines | Price per dose (2019 US$) | Vial size | Wastage rate (%) |
| --- | --- | --- | --- |
| Bacillus Calmette–Guérin (BCG) | 0.05 | 10 | 50 |
| Diphtheria, pertussis, tetanus (DPT) | 0.07 | 10 | 10 |
| Bivalent oral poliovirus vaccine (bOPV) | 0.08 | 20 | 10 |
| Measles | 0.18 | 5 | 25 |
| Hepatitis B | 0.08 | 10 | 10 |
| Pentavalent | 0.77 | 10 | 10 |
| Inactivated polio vaccine (IPV) | 1.35 | 5 | 10 |
| Rotavirus | 1.06 | 10 | 25 |
| Japanese encephalitis (JE) | 0.42 | 5 | 25 |
| Measles rubella (MR) | 0.54 | 10 | 25 |
| Pneumococcal conjugate vaccine (PCV) | 2.93 | 4 | 10 |
| Tetanus toxoid (TT) | 0.04 | 10 | 10 |
| Syringes | | | |
| 0.5 ml | 0.03 | NA | 10 |
| 5 ml | 0.02 | NA | 10 |
| 0.1 ml | 0.03 | NA | 10 |
| Source: Ministry of Health and Family Welfare, Government of India. Comprehensive multi-year plan, 2018-22. | | | |

**Table A2: Average salary of different categories of staff involved in Intensified Mission Indradhanush (IMI) Programme (US$2019)**

| Designation | Monthly gross salary |
| --- | --- |
| Chief Medical Officer of Health | 2991 |
| District Immunization Officer | 2255 |
| Additional Chief Medical Officer | 2545 |
| District Programme Manager | 611 |
| Computer Assistant for Routine Immunization (district level) | 179 |
| Medical Officer (In charge) | 1939 |
| Medical Officer | 1001 |
| Block Programme Manager | 369 |
| Block Community Mobilizer | 258 |
| Auxiliary Nurse Midwife (regular) | 609 |
| Auxiliary Nurse Midwife (contractual) | 162 |
| Cold Chain Handler | 798 |
| Health Supervisor / Health Visitor | 776 |
| Multipurpose worker | 473 |
| Data Entry Operator (block level) | 177 |

Source: Field Survey

**Table A3.1 Distribution of weighted total time cost of Intensified Mission Indradhanush (IMI) programme at the sub-centre level during October 2017 to January 2018 (US$2019)**

| District | Survey | Due list and microplan | Travel | Conducting session | Total |
| --- | --- | --- | --- | --- | --- |
| **Assam** | | | | | |
| Chirang | 3467 (24.90) | 3275 (23.53) | 364 (2.62) | 6815 (48.95) | 13922 |
| Darrang | 4300 (31.06) | 1839 (13.28) | 618 (4.46) | 7087 (51.20) | 13843 |
| Karbi Anglong | 6459 (14.49) | 7013 (15.73) | 2833 (6.36) | 28275 (63.43) | 44580 |
| Kokrajhar | 1714 (12.93) | 656 (4.95) | 1346 (10.15) | 9540 (71.96) | 13257 |
| Nagaon | 6097 (18.11) | 2179 (6.47) | 6380 (18.95) | 19014 (56.47) | 33669 |
| Average | 4407 (18.48) | 2992 (12.54) | 2308 (9.68) | 14146 (59.30) | 23854 |
| **Bihar** | | | | | |
| East Champaran | 7082 (25.42) | 2117 (7.60) | 1063 (3.82) | 17593 (63.16) | 27855 |
| Gaya | 20973 (5.56) | 2253 (0.60) | 38695 (10.26) | 315347 (83.59) | 377268 |
| Madhubani | 22552 (13.27) | 11675 (6.87) | 17926 (10.55) | 117732 (69.30) | 169886 |
| Nawada | 3563 (12.19) | 3044 (10.41) | 1869 (6.39) | 20759 (71.01) | 29234 |
| Patna | 2712 (9.87) | 1454 (5.29) | 785 (2.86) | 22520 (81.97) | 27471 |
| Sheohar | 2128 (19.77) | 505 (4.69) | 464 (4.31) | 7669 (71.23) | 10766 |
| Sitamarhi | 21957 (24.90) | 3369 (3.82) | 4423 (5.02) | 58420 (66.26) | 88169 |
| Average | 11567 (11.08) | 3488 (3.34) | 9318 (8.93) | 80006 (76.65) | 104379 |
| **Maharashtra** | | | | | |
| Ahmednagar | 13961 (30.33) | 3972 (8.63) | 935 (2.03) | 27165 (59.01) | 46033 |
| Beed | 11291 (31.15) | 3027 (8.35) | 534 (1.47) | 21400 (59.03) | 36252 |
| Jalgaon | 37210 (18.29) | 29296 (14.40) | 3097 (1.52) | 133889 (65.80) | 203491 |
| Nanded | 16834 (23.14) | 7131 (9.80) | 1042 (1.43) | 47756 (65.63) | 72763 |
| Solapur | 16717 (36.44) | 6539 (14.26) | 266 (0.58) | 22348 (48.72) | 45871 |
| Thane | 37071 (57.40) | 4898 (7.59) | 514 (0.80) | 22096 (34.22) | 64579 |
| Average | 22180 (28.38) | 9144 (11.70) | 1065 (1.36) | 45776 (58.56) | 78165 |
| **Rajasthan** | | | | | |
| Alwar | 21024 (37.28) | 9443 (16.74) | 2371 (4.20) | 23561 (41.78) | 56400 |
| Jaipur | 14147 (29.93) | 5650 (11.95) | 1111 (2.35) | 26363 (55.77) | 47272 |
| Jodhpur | 9827 (19.42) | 2602 (5.14) | 2682 (5.30) | 35494 (70.14) | 50605 |
| Pali | 2067 (7.33) | 3288 (11.66) | 2018 (7.16) | 20814 (73.84) | 28186 |
| Average | 11766 (25.79) | 5246 (11.50) | 2046 (4.48) | 26558 (58.22) | 45616 |
| **Uttar Pradesh** | | | | | |
| Bahraich | 24311 (5.53) | 30130 (6.85) | 28745 (6.54) | 356537 (81.08) | 439723 |
| Ballia | 21472 (4.01) | 56082 (10.47) | 25717 (4.80) | 432198 (80.71) | 535469 |
| Balrampur | 12093 (4.80) | 22130 (8.79) | 23069 (9.16) | 194500 (77.25) | 251792 |
| Banda | 31685 (9.68) | 23290 (7.12) | 15885 (4.85) | 256422 (78.35) | 327283 |
| Basti | 22224 (5.95) | 31770 (8.50) | 25851 (6.92) | 293889 (78.64) | 373733 |
| Chitrakoot | 10654 (10.14) | 10179 (9.69) | 11083 (10.55) | 73100 (69.61) | 105016 |
| Farrukkabad | 30865 (9.30) | 33773 (10.18) | 16054 (4.84) | 251115 (75.68) | 331808 |
| Hapur | 8253 (4.99) | 61021 (36.89) | 6906 (4.17) | 89248 (53.95) | 165427 |
| Hardoi | 38512 (6.32) | 44907 (7.37) | 37083 (6.09) | 488629 (80.22) | 609132 |
| Jaunpur | 42198 (4.68) | 204037 (22.64) | 43155 (4.79) | 611897 (67.89) | 901287 |
| Lucknow | 106428 (46.26) | 7051 (3.06) | 6804 (2.96) | 109795 (47.72) | 230078 |
| Mau | 24283 (6.32) | 44334 (11.55) | 29491 (7.68) | 285875 (74.45) | 383983 |
| Meerut | 5023 (9.21) | 8707 (15.96) | 1539 (2.82) | 39287 (72.01) | 54556 |
| Sidharthnagar | 53706 (12.43) | 49467 (11.45) | 20919 (4.84) | 307875 (71.27) | 431966 |
| Unnao | 33754 (7.45) | 45187 (9.98) | 34738 (7.67) | 339189 (74.90) | 452868 |
| Varanasi | 9964 (13.83) | 4027 (5.59) | 1655 (2.30) | 56402 (78.28) | 72048 |
| Average | 29714 (8.39) | 42256 (11.93) | 20543 (5.80) | 261622 (73.88) | 354136 |

Notes: (1) Conducting session included the record keeping time at session sites; (2) Figures in parenthesis represent percentage.

**Table A3.2 Distribution of weighted total time cost of Intensified Mission Indradhanush (IMI) programme at the sub-district (block) level during October 2017 to January 2018 (US$2019)**

| District | Training/meeting | Microplan | Social engagement | Vaccine distribution | Supervision | Report compilation | Total |
| --- | --- | --- | --- | --- | --- | --- | --- |
| **Assam** | | | | | | | |
| Chirang | 9161 (72.31) | 293 (2.31) | 0 (0.00) | 0 (0.00) | 3187 (25.15) | 29 (0.23) | 12669 |
| Darrang | 2100 (29.29) | 51 (0.71) | 0 (0.00) | 25 (0.35) | 4810 (67.07) | 185 (2.58) | 7172 |
| Karbi Anglong | 12111 (67.35) | 413 (2.30) | 0 (0.00) | 413 (2.30) | 4080 (22.69) | 963 (5.36) | 17981 |
| Kokrajhar | 8045 (68.62) | 231 (1.97) | 0 (0.00) | 0 (0.00) | 3282 (27.99) | 166 (1.42) | 11724 |
| Nagaon | 34817 (53.22) | 519 (0.79) | 0 (0.00) | 0 (0.00) | 28166 (43.05) | 1918 (2.93) | 65420 |
| Average | 13247 (57.61) | 301 (1.31) | 0 (0.00) | 88 (0.38) | 8705 (37.86) | 652 (2.84) | 22993 |
| **Bihar** | | | | | | | |
| East Champaran | 63042 (66.77) | 18831 (19.95) | 0 (0.00) | 78 (0.08) | 12246 (12.97) | 213 (0.23) | 94409 |
| Gaya | 41811 (43.95) | 3138 (3.30) | 0 (0.00) | 108 (0.11) | 49466 (52.00) | 600 (0.63) | 95123 |
| Madhubani | 53993 (32.78) | 1034 (0.63) | 0 (0.00) | 191 (0.12) | 107390 (65.19) | 2123 (1.29) | 164731 |
| Nawada | 15901 (63.85) | 177 (0.71) | 947 (3.80) | 35 (0.14) | 7385 (29.66) | 458 (1.84) | 24904 |
| Sheohar | 6620 (62.03) | 423 (3.97) | 0 (0.00) | 17 (0.16) | 3475 (32.56) | 137 (1.28) | 10672 |
| Sitamarhi | 36104 (44.54) | 351 (0.43) | 0 (0.00) | 0 (0.00) | 44085 (54.39) | 521 (0.64) | 81060 |
| Average | 36245 (46.18) | 3992 (5.09) | 158 (0.20) | 72 (0.09) | 37341 (47.58) | 675 (0.86) | 78483 |
| **Maharashtra** | | | | | | | |
| Ahmednagar | 23217 (26.56) | 853 (0.98) | 202 (0.23) | 463 (0.53) | 61449 (70.31) | 1217 (1.39) | 87401 |
| Beed | 15738 (45.31) | 415 (1.20) | 906 (2.61) | 192 (0.55) | 17265 (49.71) | 215 (0.62) | 34731 |
| Jalgaon | 35243 (26.05) | 569 (0.42) | 0 (0.00) | 815 (0.60) | 96435 (71.29) | 2207 (1.63) | 135269 |
| Nanded | 56621 (59.06) | 1831 (1.91) | 122 (0.13) | 404 (0.42) | 34228 (35.70) | 2670 (2.78) | 95877 |
| Solapur | 28094 (74.84) | 295 (0.79) | 0 (0.00) | 184 (0.49) | 8244 (21.96) | 721 (1.92) | 37539 |
| Average | 31783 (40.66) | 793 (1.01) | 246 (0.31) | 411 (0.53) | 43524 (55.68) | 1406 (1.80) | 78163 |
| **Rajasthan** | | | | | | | |
| Alwar | 39513 (63.30) | 443 (0.71) | 0 (0.00) | 0 (0.00) | 21318 (34.15) | 1146 (1.84) | 62420 |
| Jodhpur | 11047 (22.00) | 356 (0.71) | 0 (0.00) | 230 (0.46) | 37966 (75.60) | 618 (1.23) | 50216 |
| Pali | 26711 (57.40) | 379 (0.82) | 0 (0.00) | 0 (0.00) | 18468 (39.69) | 975 (2.09) | 46533 |
| Pratapgarh | 9920 (69.05) | 239 (1.66) | 0 (0.00) | 0 (0.00) | 4041 (28.13) | 168 (1.17) | 14368 |
| Udaipur | 22246 (38.91) | 553 (0.97) | 0 (0.00) | 0 (0.00) | 33568 (58.71) | 810 (1.42) | 57177 |
| Average | 21887 (47.43) | 394 (0.85) | 0 (0.00) | 46 (0.10) | 23072 (50.00) | 743 (1.61) | 46143 |
| **Uttar Pradesh** | | | | | | | |
| Bahraich | 75148 (50.26) | 815 (0.54) | 0 (0.00) | 6744 (4.51) | 62738 (41.96) | 4082 (2.73) | 149527 |
| Ballia | 64583 (36.74) | 1019 (0.58) | 821 (0.47) | 12853 (7.31) | 92510 (52.62) | 4019 (2.29) | 175805 |
| Balrampur | 17863 (35.75) | 149 (0.30) | 249 (0.50) | 7418 (14.85) | 23326 (46.69) | 957 (1.91) | 49962 |
| Banda | 30917 (40.55) | 320 (0.42) | 56 (0.07) | 4900 (6.43) | 39266 (51.51) | 778 (1.02) | 76237 |
| Basti | 45002 (36.91) | 1320 (1.08) | 412 (0.34) | 4929 (4.04) | 66092 (54.21) | 4158 (3.41) | 121912 |
| Chitrakoot | 21304 (46.53) | 108 (0.24) | 255 (0.56) | 1564 (3.42) | 21109 (46.10) | 1448 (3.16) | 45788 |
| Farrukkabad | 34462 (49.26) | 239 (0.34) | 0 (0.00) | 5941 (8.49) | 26190 (37.44) | 3125 (4.47) | 69959 |
| Hapur | 27671 (50.96) | 295 (0.54) | 352 (0.65) | 1910 (3.52) | 23587 (43.44) | 482 (0.89) | 54297 |
| Hardoi | 69264 (41.85) | 444 (0.27) | 233 (0.14) | 11989 (7.24) | 81742 (49.39) | 1823 (1.10) | 165497 |
| Jaunpur | 82180 (34.72) | 984 (0.42) | 1444 (0.61) | 15557 (6.57) | 133200 (56.27) | 3361 (1.42) | 236727 |
| Mau | 50216 (40.36) | 741 (0.60) | 0 (0.00) | 6450 (5.18) | 65386 (52.55) | 1640 (1.32) | 124433 |
| Sidharthnagar | 56908 (38.84) | 699 (0.48) | 0 (0.00) | 6931 (4.73) | 79245 (54.08) | 2738 (1.87) | 146520 |
| Unnao | 69667 (40.95) | 1190 (0.70) | 2058 (1.21) | 13949 (8.20) | 79014 (46.45) | 4237 (2.49) | 170115 |
| Average | 49630 (40.66) | 640 (0.52) | 452 (0.37) | 7780 (6.37) | 61031 (50.00) | 2527 (2.07) | 122060 |

Note: Figures in parenthesis represent percentage.

**Table A3.3 Distribution of weighted total time cost of Intensified Mission Indradhanush (IMI) programme at the district level during October 2017 to January 2018 (US$2019)**

| Districts | Training/meeting | Microplan | Social engagement | Supervision | Report compilation | Total |
| --- | --- | --- | --- | --- | --- | --- |
| **Assam** | | | | | | |
| Chirang | 2871 (61.20) | 11 (0.23) | 0 (0.00) | 1800 (38.36) | 10 (0.22) | 4692 |
| Darrang | 2545 (56.26) | 103 (2.27) | 0 (0.00) | 1814 (40.11) | 62 (1.36) | 4524 |
| Karbi Anglong | 2503 (43.74) | 20 (0.34) | 0 (0.00) | 3003 (52.47) | 197 (3.45) | 5723 |
| Kokrajhar | 3362 (50.69) | 49 (0.74) | 0 (0.00) | 3040 (45.84) | 181 (2.73) | 6631 |
| Nagaon | 3956 (54.50) | 91 (1.25) | 0 (0.00) | 3191 (43.97) | 20 (0.28) | 7259 |
| Average | 3047 (52.85) | 55 (0.95) | 0 (0.00) | 2570 (44.57) | 94 (1.63) | 5766 |
| **Bihar** | | | | | | |
| East Champaran | 5155 (50.08) | 3 (0.03) | 0 (0.00) | 5048 (49.04) | 87 (0.85) | 10293 |
| Gaya | 8173 (48.59) | 28 (0.16) | 399 (2.37) | 8155 (48.49) | 64 (0.38) | 16820 |
| Madhubani | 12912 (44.98) | 164 (0.57) | 355 (1.24) | 15259 (53.16) | 16 (0.06) | 28706 |
| Nawada | 550 (23.20) | 2 (0.10) | 0 (0.00) | 1722 (72.62) | 97 (4.07) | 2372 |
| Patna | 8498 (57.51) | 318 (2.15) | 76 (0.51) | 5454 (36.91) | 430 (2.91) | 14776 |
| Sheohar | 3510 (35.28) | 15 (0.15) | 0 (0.00) | 6415 (64.49) | 8 (0.08) | 9949 |
| Sitamarhi | 12489 (43.00) | 5 (0.02) | 0 (0.00) | 16441 (56.60) | 113 (0.39) | 29047 |
| Average | 7327 (45.81) | 76 (0.48) | 119 (0.74) | 8356 (52.24) | 117 (0.73) | 15995 |
| **Maharashtra** | | | | | | |
| Ahmednagar | 6330 (37.01) | 45 (0.26) | 0 (0.00) | 10504 (61.42) | 223 (1.30) | 17102 |
| Beed | 11746 (65.91) | 140 (0.78) | 0 (0.00) | 5370 (30.13) | 567 (3.18) | 17823 |
| Jalgaon | 5388 (56.59) | 82 (0.86) | 0 (0.00) | 3670 (38.55) | 381 (4.01) | 9521 |
| Nanded | 555 (3.74) | 209 (1.41) | 0 (0.00) | 13080 (88.13) | 999 (6.73) | 14843 |
| Solapur | 4040 (29.77) | 148 (1.09) | 0 (0.00) | 8966 (66.08) | 414 (3.05) | 13567 |
| Thane | 12724 (89.06) | 56 (0.39) | 0 (0.00) | 822 (5.75) | 685 (4.79) | 14286 |
| Average | 6797 (46.80) | 113 (0.78) | 0 (0.00) | 7069 (48.67) | 545 (3.75) | 14524 |
| **Rajasthan** | | | | | | |
| Alwar | 2601 (60.53) | 3 (0.08) | 0 (0.00) | 1647 (38.34) | 45 (1.06) | 4296 |
| Jaipur | 4466 (43.67) | 295 (2.89) | 0 (0.00) | 5349 (52.30) | 117 (1.15) | 10228 |
| Jodhpur | 1446 (28.13) | 0 (0.00) | 0 (0.00) | 2488 (48.38) | 1208 (23.49) | 5142 |
| Pali | 1882 (97.47) | 0 (0.00) | 0 (0.00) | 0 (0.00) | 49 (2.53) | 1931 |
| Pratapgarh | 731 (51.57) | 12 (0.83) | 0 (0.00) | 620 (43.68) | 56 (3.92) | 1418 |
| Udaipur | 2706 (95.30) | 88 (3.08) | 0 (0.00) | 0 (0.00) | 46 (1.62) | 2839 |
| Average | 2305 (53.50) | 66 (1.54) | 0 (0.00) | 1684 (39.08) | 254 (5.88) | 4309 |
| **Uttar Pradesh** | | | | | | |
| Bahraich | 34364 (88.79) | 33 (0.08) | 0 (0.00) | 3589 (9.27) | 718 (1.85) | 38704 |
| Ballia | 10317 (30.36) | 37 (0.11) | 0 (0.00) | 22987 (67.65) | 640 (1.88) | 33980 |
| Balrampur | 1520 (33.45) | 14 (0.32) | 333 (7.32) | 2388 (52.57) | 288 (6.33) | 4542 |
| Banda | 8544 (31.88) | 0 (0.00) | 713 (2.66) | 17519 (65.36) | 26 (0.10) | 26803 |
| Basti | 10154 (47.64) | 0 (0.00) | 0 (0.00) | 10297 (48.31) | 862 (4.04) | 21313 |
| Chitrakoot | 6840 (33.69) | 0 (0.00) | 0 (0.00) | 13392 (65.97) | 69 (0.34) | 20301 |
| Farrukkabad | 6229 (36.64) | 44 (0.26) | 0 (0.00) | 10662 (62.72) | 63 (0.37) | 16999 |
| Hapur | 8733 (52.98) | 0 (0.00) | 670 (4.06) | 6644 (40.31) | 437 (2.65) | 16484 |
| Hardoi | 11203 (48.54) | 0 (0.00) | 0 (0.00) | 11850 (51.35) | 25 (0.11) | 23079 |
| Jaunpur | 14092 (63.38) | 59 (0.27) | 0 (0.00) | 8021 (36.07) | 63 (0.28) | 22234 |
| Lucknow | 18179 (43.06) | 8 (0.02) | 0 (0.00) | 23987 (56.83) | 38 (0.09) | 42212 |
| Mau | 4376 (30.72) | 66 (0.46) | 0 (0.00) | 9738 (68.37) | 63 (0.44) | 14244 |
| Meerut | 20065 (49.55) | 169 (0.42) | 0 (0.00) | 20110 (49.66) | 149 (0.37) | 40494 |
| Sidharthnagar | 2096 (20.98) | 11 (0.11) | 0 (0.00) | 7821 (78.28) | 63 (0.63) | 9991 |
| Unnao | 13000 (52.91) | 5 (0.02) | 0 (0.00) | 11488 (46.76) | 76 (0.31) | 24569 |
| Varanasi | 15441 (25.97) | 169 (0.28) | 0 (0.00) | 43730 (73.56) | 110 (0.19) | 59451 |
| Average | 11572 (44.57) | 39 (0.15) | 107 (0.41) | 14014 (53.98) | 231 (0.89) | 25963 |

Note: Figures in parenthesis represent percentage.

**Table A4:** **Distribution of financial cost of Intensified Mission Indradhanush (IMI) programme in selected states (US$2019)**

| Districts | Microplanning | Training and meeting | Communication | Vaccine transport | Supervision | ASHA incentives | Mobile team | Sub-centre travel expenses | Vaccines | Syringes | Others | Total |
| --- | --- | --- | --- | --- | --- | --- | --- | --- | --- | --- | --- | --- |
| **Assam** | | | | | | | | | | | | |
| Chirang | 75 (0.74) | 4251 (42.04) | 529 (5.23) | 338 (3.35) | 1510 (14.93) | 728 (7.20) | 278 (2.75) | 75 (0.74) | 2004 (19.82) | 64 (0.63) | 259 (2.56) | 10112 |
| Karbi Anglong | 3982 (9.84) | 1953 (4.83) | 3936 (9.73) | 2896 (7.16) | 7632 (18.87) | 2639 (6.52) | 3818 (9.44) | 498 (1.23) | 10356 (25.60) | 344 (0.85) | 2398 (5.93) | 40453 |
| Kokrajhar | 2048 (9.50) | 1227 (5.69) | 3674 (17.05) | 506 (2.35) | 3794 (17.60_ | 2979 (13.82) | 903 (4.19) | 130 (0.60) | 5175 (24.01) | 167 (0.77) | 949 (4.40) | 21551 |
| Nagaon | 1980 (5.88) | 895 (2.66) | 1323 (3.93) | 944 (2.80) | 8279 (24.60) | 718 (2.13) | 3697 (10.98) | 4 (0.01) | 14804 (43.98) | 637 (1.89) | 379 (1.13) | 33662 |
| Average | 2021 (7.64) | 2081 (7.87) | 2366 (8.95) | 1171 (4.43) | 5304 (20.06) | 1766 (6.68) | 2174 (8.22) | 177 (0.67) | 8085 (30.57) | 303 (1.15) | 996 (3.77) | 26444 |
| **Maharashtra** | | | | | | | | | | | | |
| Ahmednagar | 0 (0.00) | 0 (0.00) | 6798 (36.79) | 810 (4.38) | 5149 (27.86) | 1620 (8.77) | 0 (0.00) | 172 (0.93) | 3683 (19.93) | 247 (1.34) | 0 (0.00) | 18478 |
| Beed | 2531 (15.99) | 217 (1.37) | 5554 (35.09) | 733 (4.63) | 752 (4.75) | 1451 (9.16) | 0 (0.00) | 34 (0.22) | 4284 (27.07) | 272 (1.72) | 0 (0.00) | 15829 |
| Jalgaon | 5120 (10.56) | 0 (0.00) | 13494 (27.83) | 955 (1.97) | 0 (0.00) | 4961 (10.23) | 0 (0.00) | 133 (0.27) | 8839 (18.23) | 499 (1.03) | 14492 (29.89) | 48492 |
| Nanded | 4533 (27.83) | 3532 (21.69) | 2798 (17.18) | 550 (3.37) | 889 (5.46) | 1085 (6.66) | 0 (0.00) | 154 (0.95) | 2568 (15.77) | 176 (1.08) | 0 (0.00) | 16285 |
| Solapur | 0 (0.00) | 0 (0.00) | 2590 (7.85) | 295 (0.89) | 6949 (21.07) | 15091 (45.76) | 0 (0.00) | 32 (0.10) | 7552 (22.90) | 471 (1.43) | 0 (0.00) | 32979 |
| Thane | 0 (0.00) | 0 (0.00) | 12406 (39.24) | 0 (0.00) | 1837 (5.81) | 3993 (12.63) | 0 (0.00) | 125 (0.40) | 12576 (39.78) | 679 (2.15) | 0 (0.00) | 31616 |
| Average | 2031 (7.44) | 625 (2.29) | 7273 (26.66) | 557 (2.04) | 2596 (9.52) | 4700 (17.23) | 0 (0.00) | 108 (0.40) | 6584 (24.13) | 391 (1.43) | 2415 (8.85) | 27280 |
| **Uttar Pradesh** | | | | | | | | | | | | |
| Bahraich | 3020 (0.73) | 0 (0.00) | 30215 (7.28) | 8626 (8626) | 12304 (2.96) | 15713 (3.78) | 0 (0.00) | 4204 (1.01) | 330271 (79.53) | 10951 (2.64) | 0 (0.00) | 415304 |
| Ballia | 781 (0.58) | 0 (0.00) | 0 (0.00) | 11596 (8.55) | 6442 (4.75) | 19964 (14.72) | 0 (0.00) | 504 (0.37) | 91538 (67.51) | 4770 (3.52) | 0 (0.00) | 135594 |
| Balrampur | 5134 (2.53) | 289 (0.14) | 10666 (5.26) | 8344 (4.12) | 14012 (6.92) | 10001 (4.94) | 0 (0.00) | 6105 (3.01) | 140166 (69.19) | 4693 (2.32) | 3172 (1.57) | 202583 |
| Banda | 4420 (4.87) | 386 (0.42) | 0 (0.00) | 6694 (7.37) | 2831 (3.12) | 5020 (5.53) | 0 (0.00) | 938 (1.03) | 50047 (55.09) | 2458 (2.71) | 18051 (19.87) | 90845 |
| Basti | 3811 (3.38) | 802 (0.71) | 0 (0.00) | 11777 (10.43) | 6241 (5.53) | 16627 (14.73) | 0 (0.00) | 1827 (1.62) | 66592 (58.99) | 3575 (3.17) | 1632 (1.45) | 112884 |
| Chitrakoot | 1055 (2.27) | 0 (0.00) | 3171 (6.82) | 1893 (4.07) | 3261 (7.01) | 2916 (6.27) | 0 (0.00) | 326 (0.70) | 27434 (58.96) | 1338 (2.87) | 5134 (11.04) | 46527 |
| Farrukkabad | 347 (0.32) | 25 (0.02) | 5590 (5.11) | 6237 (5.70) | 3617 (3.31) | 10800 (9.87) | 1059 (0.97) | 1520 (1.39) | 76308 (69.73) | 3855 (3.52) | 68 (0.06) | 109427 |
| Hapur | 260 (0.56) | 0 (0.00) | 0 (0.00) | 3187 (6.84) | 2125 (4.56) | 6374 (13.69) | 0 (0.00) | 1172 (2.52) | 31800 (68.30) | 1643 (3.53) | 0 (0.00) | 46562 |
| Hardoi | 955 (0.52) | 1015 (0.56) | 2736 (1.50) | 6002 (3.29) | 11009 (6.03) | 15230 (8.35) | 0 (0.00) | 2090 (1.15) | 136652 (74.88) | 6805 (3.73) | 0 (0.00) | 182496 |
| Jaunpur | 6866 (2.59) | 29461 (11.11) | 972 (0.37) | 11048 (4.16) | 26804 (10.10) | 22096 (8.33) | 694 (0.26) | 967 (0.36) | 158076 (59.59) | 8288 (3.12) | 0 (0.00) | 265272 |
| Lucknow | 2449 (2.15) | 0 (0.00) | 11668 (10.26) | 6419 (5.65) | 12414 (10.92) | 12839 (11.29) | 0 (0.00) | 6026 (5.30) | 58855 (51.76) | 3042 (2.68) | 0 (0.00) | 113712 |
| Mau | 3749 (3.25) | 2131 (1.85) | 8237 (7.14) | 7107 (6.16) | 14047 (12.17) | 13008 (11.27) | 0 (0.00) | 272 (0.24) | 60221 (52.17) | 3530 (3.06) | 3136 (2.72) | 115438 |
| Meerut | 1536 (2.49) | 0 (0.00) | 3998 (6.47) | 2984 (4.83) | 6281 (10.17) | 6077 (9.84) | 0 (0.00) | 1528 (2.48) | 37528 (60.78) | 1814 (2.94) | 0 (0.00) | 61745 |
| Sidharthnagar | 752 (0.36) | 0 (0.00) | 1143 (0.54) | 12149 (5.78) | 8479 (4.04) | 19135 (9.11) | 0 (0.00) | 2499 (1.19) | 160579 (76.44) | 5329 (2.54) | 0 (0.00) | 210066 |
| Unnao | 781 (0.49) | 0 (0.00) | 4369 (2.77) | 10557 (6.68) | 21625 (13.69) | 17278 (10.94) | 0 (0.00) | 5756 (3.64) | 92807 (58.74) | 4821 (3.05) | 0 (0.00) | 157993 |
| Varanasi | 1707 (2.61) | 0 (0.00) | 6040 (9.23) | 3094 (4.73) | 6439 (9.84) | 6085 (9.30) | 0 (0.00) | 551 (0.84) | 39017 (59.60) | 1899 (2.90) | 636 (0.97) | 65467 |
| Average | 2351 (1.61) | 2132 (1.46) | 5550 (3.81) | 7357 (5.05) | 9871 (6.77) | 12448 (8.54) | 110 (0.08) | 2268 (1.56) | 97368 (66.81) | 4301 (2.95) | 1989 (1.36) | 145745 |

Notes: (1) Microplanning includes line listing; vaccine transport includes payments for alternate vaccine delivery (AVD); supervision includes mobility support. (2) Figures in parenthesis represent percentage
